# Geometric description of self-interaction potential in symmetric protein complexes

**DOI:** 10.1038/s41597-019-0058-x

**Published:** 2019-05-17

**Authors:** Charly Empereur-Mot, Hector Garcia-Seisdedos, Nadav Elad, Sucharita Dey, Emmanuel D. Levy

**Affiliations:** 1 Department of Structural Biology, Weizmann Institute of Science, Rehovot, 7610001 Israel; 2 Department of Innovative Technologies, University of Applied Sciences and Arts of Southern Switzerland, Manno, 6928 Switzerland; 3 Department of Chemical Research Support, Weizmann Institute of Science, Rehovot, 7610001 Israel

**Keywords:** Protein analysis, Protein structure predictions, X-ray crystallography

## Abstract

Proteins can self-associate with copies of themselves to form symmetric complexes called homomers. Homomers are widespread in all kingdoms of life and allow for unique geometric and functional properties, as reflected in viral capsids or allostery. Once a protein forms a homomer, however, its internal symmetry can compound the effect of point mutations and trigger uncontrolled self-assembly into high-order structures. We identified mutation hot spots for supramolecular assembly, which are predictable by geometry. Here, we present a dataset of descriptors that characterize these hot spot positions both geometrically and chemically, as well as computer scripts allowing the calculation and visualization of these properties for homomers of choice. Since the biological relevance of homomers is not readily available from their X-ray crystallographic structure, we also provide reliability estimates obtained by methods we recently developed. These data have implications in the study of disease-causing mutations, protein evolution and can be exploited in the design of biomaterials.

## Abstract (summary)

**Table:** 

Design Type(s)	protein interaction analysis objective • protein structure prediction objective • modeling and simulation objective
Measurement Type(s)	protein complex
Technology Type(s)	computational modeling technique
Factor Type(s)	Filtering • source
Sample Characteristic(s)	laboratory environment

Machine-accessible metadata file describing the reported data (ISA-Tab format)

## Background & Summary

The controlled association of proteins into functional complexes is central to the myriad of biochemical processes required to maintain cellular functions^1,2^. The symmetry of protein complexes enables unique biological properties: compact genetic encoding of large assemblies such as viral capsids, cytoskeleton tubules and filaments, or cooperative, switch-like transitions involving allostery. However, we recently observed that the repetition of subunits within homomers can exacerbate the effect of point mutations, resulting in the homomer’s uncontrolled self-assembly^3^.

For a new mode of protein assembly to take place, a new interaction must be created. Previous work showed that the chemical composition of protein interfaces, although distinct from surfaces, is relatively close. Indeed, two amino-acid substitutions are sufficient, on average, to shift the chemical composition of a protein surface patch into that of an interface^4^, suggesting that point mutations may frequently trigger new interactions, as in the sickle-cell disease^5^.

Here, we need to distinguish homotypic interactions, where two identical parts of the structure are in contact, from heterotypic interaction, where two distinct structural parts are in contact. Homotypic interactions are more frequently sampled by chance than heterotypic interactions are^6–8^. When occurring at the surface of a monomer or at the surface of a cyclic complex, a new homotypic interaction will likely yield a finite dimerization event^9–11^. However, among homomers with dihedral symmetry, the emergence of a new self-interaction necessarily triggers an infinite (open) self-assembly^9^.

In our previous work^3^, we introduced point mutations solely designed to increase surface hydrophobicity into 12 dihedral homomers from *Escherichia coli*. Remarkably, these mutations triggered new self-interactions resulting in all complexes forming high-order supramolecular assemblies both *in vitro* and *in vivo* upon heterologous expression in *Saccharomyces cerevisiae*. Structural examination of these mutants allowed us to identify a novel descriptor: the normal distance to the closest bounding plane (nDp) of a symmetric oligomer, which describes a residue’s position on the global quaternary structure. The lower the nDp, the closer the amino acid is to the apex or “tip” of the assembly along a symmetry axis, and the more its mutation has the potential to trigger the formation of a high-order assembly^3^.

Accordingly, we then showed that the greater potential of these geometric hot spots to trigger assemblies was counterbalanced chemically by an enrichment in hydrophilic amino acids^3^. We measured the interaction propensity of surface regions on 1,990 dihedral homomers of known structure using the ‘stickiness’ scale introduced by Levy *et al*.^3,12^ and detailed below. Our results indicated that surface regions with high potential to trigger supramolecular assemblies upon mutation (i.e. low nDp) counterbalanced this risk by residues with low interaction propensity, or stickiness^3^.

Here, we present a dataset of descriptors that characterize these geometric hot spot positions and buffering effects on 165,916 proposed biological assemblies from the Protein Data Bank (PDB)^13,14^, together with the workflow and computer scripts used to compute these descriptors^15^.

These data serve multiple uses: (i) they will be important to consider in future studies predicting the molecular consequences of mutations, including single nucleotide polymorphisms, (ii) from an evolutionary standpoint, they describe molecular phenotypes that may constrain amino acid changes and thereby, could be considered in phylogenetic models of sequence evolution, and (iii) in the field of bio-materials design, these data facilitate the application of our simple strategy to program protein self-assembly at length scales up to several micrometers either *in vitro* or *in vivo*, using the PDB as a source of natural “building blocks”.

## Methods

### Normal distance to the closest bounding plane (nDp) calculation

To study the effects of point mutations on symmetric homomers, we defined a novel structural descriptor based on quaternary structure geometry. We called this descriptor the “normal distance to the closest bounding plane”, or nDp. These methods are expanded versions of descriptions in our related work^3^.

**Fig. 1:**
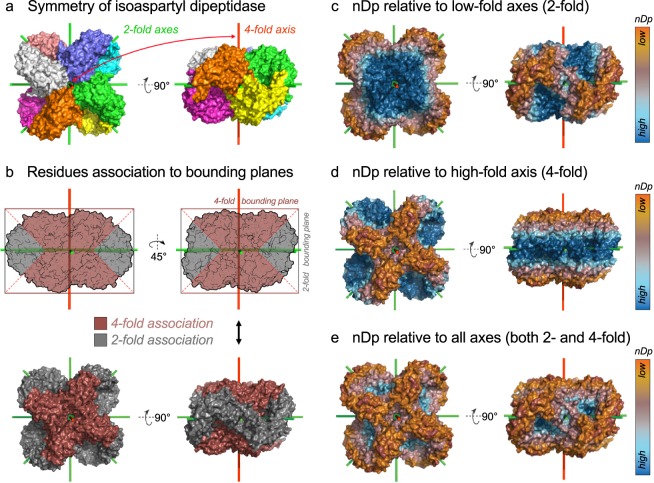
Principle of calculation of different versions of the normal distance to the closest bounding plane (nDp) visualized on the dihedral structure of isoaspartyl dipeptidase. (**a**) Coloration of the biological assembly of isoaspartyl dipeptidase by subunits (PDB accession 1POK^35^). Symmetry axes appear in green (2-fold axes) and red (4-fold axis). (**b**) Residues are assigned to their closest bounding plane. For this D4 complex, bounding planes originate from either 2- or 4-fold axes (grey and brown, respectively). (**c**) Visualization of the nDp-2-fold. (**d**) Visualization of the nDp-n-fold, where n = 4 in the case of this D4 complex. (**e**) Visualization of the nDp, which is relative to all bounding planes of the assembly independently of axes folds.

We reasoned that for point mutations to act synergistically in the creation of novel self-interacting interfaces, the affected residues at the surface of one copy of the homomer must be altogether accessible to the surface of other copies of this oligomer. Bounding planes, which are orthogonal to symmetry axes, capture such information. The nDp measure thus describes the distance of a residue from the closest apex of a quaternary structure along a symmetry axis (Fig. 1). The lower a residue’s nDp, the higher its potential to mediate interactions with another copy of the homomer, and the more likely it is to trigger a novel self-interacting interface upon mutation^3^. To calculate the nDp, a symmetry axis is considered as a unit (1 Å) vector **s** originating from the center of mass of the assembly. Similarly, the Cα of each residue *i* defines a vector **r**_*i*_ originating from the center of mass. For each symmetry axis *a* of the assembly, two bounding planes parallel to one another are defined. They are orthogonal to the symmetry axis considered, and intersect at the maximal (d_*a*,*max*_) and minimal (d_*a*,*min*_) values of the dot product **s** · **r**_*i*_, considering all residues *i* of the quaternary structure. The measure nDp for a given residue *i* is calculated with respect to a particular axis as the minimal distance to either of its bounding planes *a*, as follows: nDp_*a*,*i*_ = min(d_*a*,*max*_ − **s** · **r**_*i*_, **s** · **r**_*i*_ − d_*a*,*min*_)^7^.

Among cyclic complexes, which have a single axis of symmetry, there is no ambiguity to calculate nDp with the formula above. However, homomers with dihedral symmetry have multiple axes of symmetry, so multiple nDp values can be computed for each residue (one for each symmetry axis). Here, we consider three cases:

(i) nDp relative to bounding planes originating from 2-fold axes, where each residue is assigned the lowest nDp value relative to all 2-fold axes (i.e. nDp-low-fold or nDp-2-fold, Fig. 1c),
(ii) nDp relative to bounding planes originating from the n-fold axis (i.e. nDp-high-fold or nDp-n-fold, Fig. 1d), and
(iii) nDp relative to all bounding planes originating from all axes, whereby each residue is assigned the lowest nDp value relative to all axes (i.e. nDp, Fig. 1e). In our previous study^3^, we employed this definition.

Importantly, D2 homomers have three 2-fold axes and so it is not possible to distinguish between axes’ folds. Thus, for those we only employ nDp definition number 3.

### Environment stickiness calculation

In our previous work, we observed that regions with high geometric potential to trigger self-assembly counterbalanced that potential by negative design consisting of a lower than average chemical potential for self-assembly. We measured the chemical potential for self-assembly of a given surface patch by the “stickiness” of amino acids it contains, introduced in our previous work^12^ and described in detail below.

**Fig. 2:**
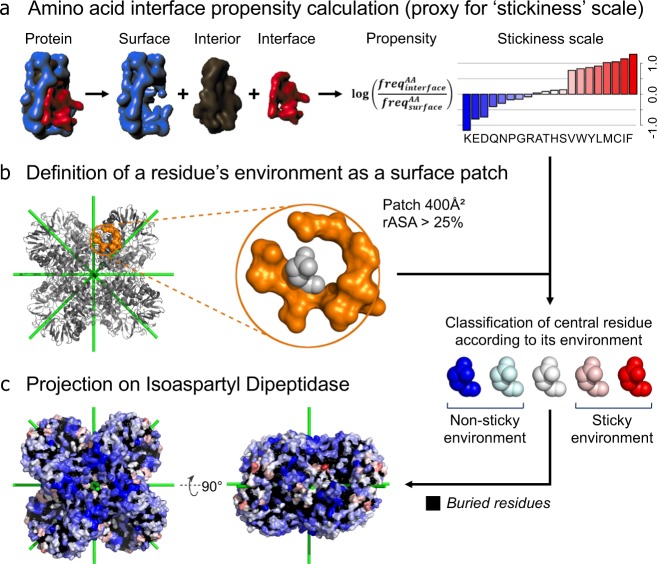
Workflow used to calculate the ‘environment stickiness’ of a residue illustrated on the dihedral structure of isoaspartyl dipeptidase (PDB accession 1POK). (**a**) Calculation of the ‘stickiness’ scale. Surface and interface regions are defined for each protein of the dataset^4^. The stickiness of an amino acid is then defined as the log-ratio of its frequency at protein-protein interfaces relative to solvent-exposed surfaces^12^. (**b**) The environment of a residue of interest is defined by surface residues within a 400 Å^2^ patch centered on the Cα of the residue of interest^12^. The central residue is excluded from the calculation. (**c**) Projection of the environment stickiness on isoaspartyl dipeptidase. Residues protected by low interaction propensity environments appear in blue.

The stickiness of an amino acid is defined as the log-ratio of its frequency at protein-protein interfaces relative to solvent-exposed surfaces (Fig. 2a). The stickiness scale thus quantifies the trade-off between the probabilities of finding a given amino acid involved in an interaction with another protein versus being in a solvated environment (Fig. 2a)^12^. Its calculation is based on a set of 397 non-redundant protein structures from *E*. *coli*. Surface and interface protein regions were defined using the residues relative accessible solvent area in the complexed and unbound states (rASAc and rASAu, respectively)^4,12^. If a residue has a rASAc value superior to 25% and the delta between rASAc and rASAu is null, then this residue is assigned to the surface (ΔrASA = 0 & rASAc > 25%). Interface residues were defined as those belonging to the interface core (ΔrASA > 0 & rASAc < 25% & rASAu > 25%). The stickiness scale employed here is based on *E*. *coli* proteins, but it is robust to using different sets of proteins. For example, deriving stickiness scales based on proteins from *S*. *cerevisiae* and *H*. *sapiens* showed high correlation values (*R*_*E*. *coli*−*S*. *cere*_ = 0.94, *R*_*E*. *coli*−*S*. *sapi*_ = 0.97)^12^.

Next, the ‘environment stickiness’ of a residue of interest is calculated based on its surrounding surface residues, by averaging their stickiness values (Fig. 2b)^12^. The residue at the center of the patch is excluded since we focus on quantifying the buffering effects in the residue’s vicinity. The reasoning behind this approach is that residues in more sticky environments are expected to have a higher probability of triggering protein-protein interfaces upon mutation to more sticky or more hydrophobic residues^12^. Surrounding surface residues are defined as those whose Cα is located within a 400 Å^2^ patch centered on the Cα of the residue of interest (i.e. a maximum Cα-Cα distance of 11.28 Å). The surface region defined for the environment stickiness calculation are associated to a rASAc > 25%, without considering any delta between rASAc and rASAu. All buried residues (rASAc < 25%) are ignored and no stickiness is computed for those.

### Biological relevance of homomers

The biologically relevant quaternary structure (QS) of a protein is not readily available from its X-ray crystallographic structure, which provides the atomic coordinates of the asymmetric unit (ASU) only. Indeed, the QS may be formed by parts of several ASUs or be a sub-part of one ASU. The challenge is, therefore, to distinguish fortuitous crystal contacts from biological ones forming the QS^16,17^. Numerous approaches such as PISA^18^ and EPPIC^19^ have been developed to predict QS information from X-ray crystallographic structures. In this dataset we provide predictions based on the integration of PISA and EPPIC approaches together with novel ones we recently developed, named QSalign/anti-QSalign and QSbio^20^. These methods are summarized from descriptions in our related work^20^.

QSalign employs evolutionary conservation of quaternary structure geometry as evidence of biological significance^20^. Quaternary structure conservation is inferred following the structural superposition of full homomers using Kpax^21^ and is quantified by a multichain version of the TM-score^22^. Anti-QSalign takes a complementary approach where the absence of QS of homologues is predictive of a monomeric state.

**Fig. 3:**
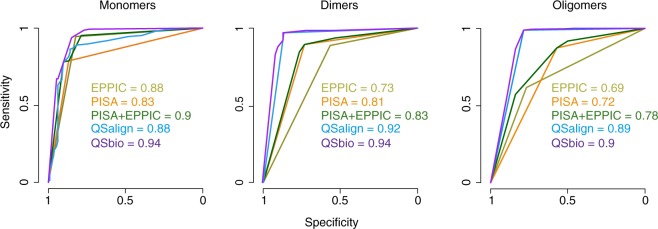
Benchmark of individual methods and of their integration into QSbio. ROC curves are shown for each method with their respective area under the curve (AUC) values; separately for monomers, dimers and larger oligomers. The benchmark was carried out as earlier^20^, using the manually curated PiQSi database as a gold-standard dataset.

Lastly, QSbio scores the relevance of a QS based on the predictions from three methods (PISA^18^, EPPIC^19^, QSalign/anti-QSalign^20^) and provides a confidence estimate per assembly in the form of a probability for the QS to be incorrect^20^. Those probabilities are estimated based on a benchmark (Fig. 3), and are given in the table of assemblies descriptors (protein_assemblies_description.csv.tar.gz^15^).

### Other descriptors acquisition

Assemblies descriptors were retrieved from the 3DComplex database^23^: number of subunits, molecular weight, resolution, symmetry types, symmetry axes and Uniprot^24^ accession codes (protein_assemblies_description.csv.tar.gz^15^). Regarding residue descriptors, absolute and relative accessible surface area (ASA) were calculated using CCP4^25^ Areaimol^26,27^. Relative ASA values initially superior to 100 were corrected to 100. For convenience, stickiness scale values from Levy *et al*.^12^ were also included for each residue entry.

### Datasets construction

As a starting point to build the datasets of assemblies we present in this paper, we interrogated the 3DComplex database^23^ to retrieve assemblies that: (i) do not break into separated sub-structures when ignoring subunit-subunit contacts of less than 5 residues per chain on average, (ii) have at least one domain defined in either SCOP^28^, Pfam^28,29^ or ECOD^30^, (iii) do not contain superposed chains, and (iv) do not exclusively contain Cα information (low resolution structures). This process allowed us to retrieve 165,916 proposed biological assemblies from the PDB^13^ for which all descriptors cited in this study are provided^15^.

## Data Records

### Datasets description

Data are split into 6 tables containing 3 different types of information: assemblies descriptors, assemblies symmetry axes coordinates or residue descriptors (Table 1). All data and scripts are available on figshare at: 10.6084/m9.figshare.6586958.v2^15^. We provide residue descriptors as 4 tables that regroup either symmetric or asymmetric protein structures (‘sym’ and ‘asym’ indicators, respectively) for either all 165,916 assemblies retrieved from the PDB^13^ or their non-redundant subset of 40,109 assemblies (‘all’ and ‘h80’ indicators, respectively). To facilitate data loading and manipulation, table ‘residues_h80_sym_protein_assemblies’ is a non-redundant subset of table ‘residues_all_sym_protein_assemblies’ and table ‘residues_h80_asym_protein_assemblies’ is a non-redundant subset of table ‘residues_all_asym_protein_assemblies’. Non-redundant subsets were derived from 3DComplex^23^. This process eliminates proteins that share the same domain architecture as defined in SCOP^28^, Pfam^29^ or ECOD^30^ and more than 80% sequence identity. Importantly, the quaternary structure is taken into account when filtering redundant structures, so different quaternary structures sharing the same sequence are kept.

**Table 1:** Overview of tables content.

Table name	Content	Nb assemblies	Rows	Cols	File size
protein_assemblies_description	Assemblies descriptors	165,916	165,916	13	8.8 Mb
protein_assemblies_symmetry_axes	Axes coordinates	69,191	105,965	5	3.2 Mb
residues_all_sym_protein_assemblies	Residue descriptors	69,922	56,547,328	17	4.329 Gb
residues_all_asym_protein_assemblies	Residue descriptors	95,994	32,035,629	14	2.145 Gb
residues_h80_sym_protein_assemblies	Residue descriptors	20,820	16,649,091	17	1.278 Gb
residues_h80_asym_protein_assemblies	Residue descriptors	19,289	7,024,731	14	468.7 Mb

File sizes are for uncompressed tables. Although the data present in tables ‘residues_h80_sym_protein_assemblies’ and ‘residues_h80_asym_protein_assemblies’ are subsets of tables ‘residues_all_sym_protein_assemblies’ and ‘residues_all_asym_protein_assemblies’, respectively, we decided to provide separate tables for non-redundant assemblies to facilitate data loading and manipulation.

### Assembly descriptors

**Fig. 4:**
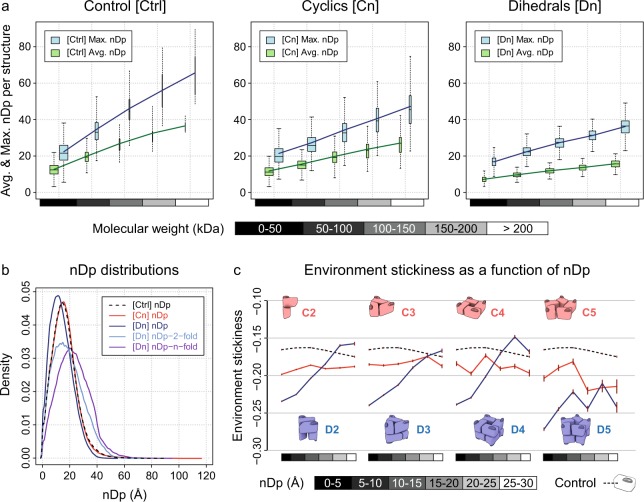
Relating the normal distance to the closest bounding plane (nDp) to assemblies’ molecular weight and environment stickiness. (**a**) Average and maximum nDp per assembly as a function of its molecular weight for control (Ctrl), cyclic (Cn) and dihedral (Dn) complexes. The control structures are monomers. Number of assemblies: (Ctrl) 11,092, (Cn) 9,996 and (Dn) 3,286. Number of residues: (Ctrl) 3,126,485, (Cn) 5,725,566 and (Dn) 4,585,996. Lines show the average per binned sample. Boxes height represents Q_1_–Q_3_ quartiles. Lower and upper hinges extend boxes by 150% of the Q_1_–Q_3_ interquartile range, in the limit of existing data. Boxes widths are proportional to the square root of sample size ratio. (**b**) Distributions of the nDp across symmetry types: control (Ctrl), cyclic (Cn) and dihedral (Dn) complexes. Number of assemblies: same as (**a**) and (Dn nDp-2-fold & Dn nDp-n-fold) 1,133. Number of residues: same as (**a**) and (Dn nDp-2-fold & Dn nDp-n-fold) 2,072,956. (**c**) Environment stickiness as a function of nDp for control (dashed lines), cyclic (red) and dihedral (blue) complexes. In accordance with our previous results^3^, environment stickiness is tuned as a function of nDp in dihedral complexes, but not in cyclic complexes. Brown error bars correspond to two standard errors. Number of assemblies: (Ctrl) 10,637, (C2) 8,626, (C3) 857, (C4) 126, (C5) 58, (D2) 2,106, (D3) 693, (D4) 282, (D5) 68. Number of residues: (Ctrl) 1,437,486, (C2) 2,018,957, (C3) 253,493, (C4) 52,484, (C5) 18,381, (D2) 910,575, (D3) 398,613, (D4) 224,067, (D5) 51,004.

Table ‘protein_assemblies_description’ stores general assemblies descriptors (Table 2). Each line corresponds to one of the 165,916 assemblies of the complete dataset. Fields ‘h_80’, ‘h_90’ and ‘tv_sticky_discard’ are binary values (0/1) indicating, respectively, whether the assembly belongs to a non-redundant subset using either a 80% sequence identity threshold, a 90% sequence identity threshold, and whether it was ignored to perform technical validation (see section “Technical Validation”, Fig. 4c).

**Table 2:** Assembly descriptors records.

Field	Description	Type
pdb_long	Four characters PDB accession code, followed by the assembly number	string
pdb_short	Four characters PDB accession code	string
uniprot	Uniprot accession code	string
resol	X-ray crystallography resolution (Å)	float
sym	Symmetry of protein assembly	string
nsub	Number of subunits in protein assembly	int
mw	Molecular weight of protein assembly (Da)	float
PiQSi	Quaternary structure validity inferred in the manually curated database PiQSi (YES/NO & PROBYES/PROBNOT). YES/PROBYES indicates likely errors.	string
QSalign	Quaternary structure validity inferred from QSalign (YES/NO & PROBYES/PROBNOT). YES/PROBYES indicates likely errors.	string
QSbio	Quaternary structure error probability from QSbio (range 0-100)	float
tv_discard	Assembly ignored in the technical validation (binary)	int
h_80	Assembly belonging to a non-redundant dataset where no two structures share the same QS and sequence identity >80% (binary)	int
h_90	Assembly belonging to a non-redundant dataset where no two structures share the same QS and sequence identity >90% (binary)	int

Each line of table protein_assemblies_description.csv.tar.gz^15^ corresponds to one unique assembly.

### Assembly symmetry axes coordinates

Information on symmetry axes coordinates is stored in table protein_assemblies_symmetry_axes.csv.tar.gz^15^ (Table 3). Each line corresponds to one symmetry axis that belongs to one of the 69,922 symmetric assemblies in the datasets, minus 731 assemblies for which symmetry axes were incorrect.

**Table 3:** Assembly symmetry axes records.

Field	Description	Type
pdb_long	Four characters PDB accession code, followed by the biological assembly number	string
fold	Symmetry axis fold	int
x	Symmetry axis unit vector orientation (x-axis)	float
y	Symmetry axis unit vector orientation (y-axis)	float
z	Symmetry axis unit vector orientation (z-axis)	float

Each line of table protein_assemblies_symmetry_axes.csv.tar.gz^15^ corresponds to one unique symmetry axis.

### Residue descriptors

Tables ‘residues_all_sym_protein_assemblies’, ‘residues_all_asym_protein_assemblies’, ‘residues_h80_sym_protein_assemblies’ and ‘residues_h80_asym_protein_assemblies’ store residue descriptors (Table 4)^15^. Each line corresponds to one unique residue of a structure’s assembly. Please note that descriptor ‘nDp’ is defined for monomers in tables ‘residues_all_asym_protein_assemblies’ and ‘residues_h80_asym_protein_assemblies’ only because we use it as a control after having generated one random symmetry axis per monomer (see section “Technical Validation”). Otherwise, calculating any nDp version on asymmetrical structures is irrelevant because it directly depends on the symmetry axes of an assembly.

**Table 4:** Residue descriptors records.

Field	Description	Type
pdb_long	Four characters PDB accession code, followed by the biological assembly number	string
chain	Protein chain in PDB file	char
num	Residue number in PDB file	int
name	Residue 3 characters code	string
letter	Residue 1 character code	char
x	Residue Cα position (x-axis)	float
y	Residue Cα position (y-axis)	float
z	Residue Cα position (z-axis)	float
rASA_in_BU	Residue relative ASA considering the complexed protein state	float
rASA_alone	Residue relative ASA considering the unbound protein state	float
sticky_scale	Residue stickiness value	float
sticky_patch	Residue environment stickiness	float
patch_size	Number of residues used for environment stickiness calculation	int
(*) nDp	Residue nDp (minimum values across all axes)	float
(**) fold	Symmetry type (2-fold, 3-fold, etc) of the axis with respect to which nDp is calculated	int
(**) nDp_n_fold	Residue nDp-n-fold	float
(**) nDp_2_fold	Residue nDp-2-fold (minimum values across all 2-fold axes)	float

Each line of tables ‘residues_all_sym_protein_assemblies’, ‘residues_all_asym_protein_assemblies’, ‘residues_h80_sym_protein_assemblies’ and ‘residues_h80_asym_protein_assemblies’^15^ corresponds to one unique residue. (*) Descriptor defined for monomers in tables ‘residues_all_asym_protein_assemblies’ and ‘residues_h80_asym_protein_assemblies’ only because we use it as a control (see section “Technical Validation”). (**) Descriptors exclusively related to high-order dihedral complexes (Dn, n > 2) and present only in tables residues_all_sym_protein_assemblies’ and ‘residues_h80_sym_protein_assemblies’.

## Technical Validation

Both manual inspection of individual examples as well as global analyses were performed to ensure the validity of the data. All residue descriptors were projected onto a few hundred assemblies for visual inspection using PyMol^31^. Measurement tools then allowed for manual validation of different nDp versions and environment stickiness calculations on several randomly selected residues. While recalculating data to provide updated datasets, we added a negative control by generating hypothetical nDp values on monomers. We used a single randomly oriented axis passing through the centroid of each monomer structure, and calculated nDp values as for any self-assembling high-order structure using this single axis (see Methods). This technical validation was performed using the non-redundant subsets of assemblies exclusively: tables ‘residues_h80_sym_protein_assemblies’ and ‘residues_h80_asym_protein_assemblies’ and ignoring assemblies for which the QS error probability calculated by QSbio was very high, i.e. above 50%.

As expected, the average and maximum nDp per assembly increase linearly with assemblies’ molecular weight, regardless of symmetry types and including control (Fig. 4a). Since all structures are considered in terms of biological assemblies, the average and maximum nDp per assembly within a given range of molecular weight is superior for control and cyclic complexes compared to dihedral complexes (Fig. 4a). This is due to dihedral complexes having at least 3 orthogonal symmetry axes (i.e. at least 6 bounding planes), whereas control and cyclic complexes only have 1 symmetry axis (i.e. 2 bounding planes). Considering the different nDp definitions at residue level, distributions are very similar regardless of protein symmetry types (Fig. 4b). Only the distributions of the nDp-2-fold and nDp-n-fold, which are exclusively calculated on high-order dihedral complexes (Dn, n > 2), are slightly more spread due to the high molecular weights of these assemblies (Fig. 4b). The distribution of the nDp-n-fold is also shifted towards higher values, since high-order dihedral complexes tend to be wider along their 2-fold axes, as for isoaspartyl dipeptidase (Fig. 1b).

Finally, we observed residues’ environment stickiness as a function of nDp across different symmetry types (Fig. 4c) and validated our previous computational results: surface regions with high potential to trigger supramolecular assemblies upon mutation (i.e. low nDp) counterbalance this risk by residues with low interaction propensity (i.e. stickiness)^7^. Environment stickiness is tuned as a function of nDp in dihedral complexes, but not in cyclic complexes nor in control (Fig. 4c). To avoid biases due to membrane and viral proteins when analysing surface stickiness, we discarded all assemblies containing one of the following chains of characters in their title, description or function PDB fields: ‘lipid’, ‘transport’, ‘rhodopsin’, ‘membran’, ‘virus’, ‘viral’. We also excluded from the technical validation those assemblies with a high probability to be non-biological (>50%).

## ISA-Tab metadata file

Download metadata file

## Data Availability

Perl scripts we used to calculate residues’ environment stickiness and different nDp versions are provided (Scripts.tar.gz^15^). Accordingly, the scripts archive contains two folders with demonstration input and output files for each script. A wrapper allows to run all calculations from a PDB file. The PyMol^31^ script we used to visualize results on protein structures is also provided. All scripts were extensively commented and made easily readable to facilitate re-use and adaptation (Table 5).

**Table 5:** Overview of the scripts archive content.

Folder	File	Type	Description
.	README.txt	README file	README file
	1pok_3.pdb	Demo Input	Demonstration PDB file
	pymol_visualization.py	PyMol Script	Enables the visualization of properties on structures, and also symmetry axes
	freesasa-2.0.3.tar	Archive	FreeSASA software v2.0.3 that needs to be installed to perform ASA calculations
	wrapper_nDp_and_stickiness_calculations.pl	Perl Script	Calculation of the different nDp versions and environment stickiness
	1pok_3.nDp_and_stickiness	Demo Output	Different nDp versions and environment stickiness for 1pok_3 in tabulated file
./nDp	nDp_calculation.pl	Perl Script	Calculation of the different nDp versions
	ananas_linux	Binary	AnAnaS software v0.6 for Linux platforms required to perform symmetry calculations, no installation is required
	ananas_mac	Binary	AnAnaS software v0.6 for Darwin (Mac) platforms required to perform symmetry calculations, no installation is required
	1pok_3.sym	Demo Output	Symmetry order and axes coordinates for 1pok_3 in tabulated file, as calculated by the software AnAnaS
	1pok_3.nDp	Demo Output	Different nDp versions for 1pok_3 in tabulated file
./environment_stickiness	environment_stickiness_calculation.pl	Perl Script	Calculation of the environment stickiness
	1pok_3.asa	Demo Output	ASA for 1pok_3 in tabulated file, as calculated by the software FreeASA
	1pok_3.stickiness	Demo Output	Environment stickiness for 1pok_3 in tabulated file

Demonstration input and output files are provided for each script. Overview of the scripts archive content. Demonstration input and output files are provided for each script. An installation of the software FreeSASA^32^ is required to run environment stickiness calculations. FreeSASA provides ASA calculations our script relies on. FreeSASA is available under MIT license and v2.0.3 is included in the scripts archive. The software AnAnaS^33,34^ (Analytical Analyzer of Symmetries) is used to detect symmetry order and symmetry axes positions required to run nDp calculations. AnAnaS is free for academic use and included in the scripts archive as a binary file.
